# Supplementary material for: Lithium systematics in global arc magmas and the importance of crustal thickening for lithium enrichment
Source: Nat Commun. 2020 Oct 20;11:5313. doi: 10.1038/s41467-020-19106-z (PMC7575555; doi:10.1038/s41467-020-19106-z)
Supplement: Supplementary file 1 — Supplementary Information [file 41467_2020_19106_MOESM1_ESM.pdf]

Lithium systematics in global arc magmas and the importance of crustal thickening for lithium enrichment

Chen et al.

Supplementary Information

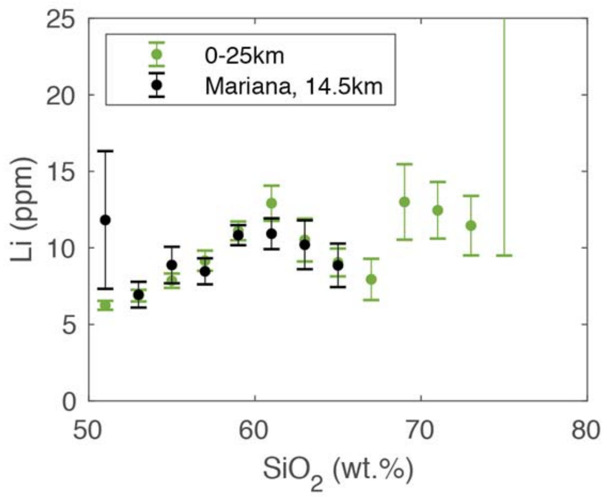

Supplementary Figure 1. Comparison of Li differentiation trends of the Mariana arc with grouped thin arcs. The error bars are 2se. There are anomalously high Li signatures from primitive Mariana arc basalts with big errors, which results from the extensive submarine alteration of basaltic rocks dredged from the West Mariana basin[1,2]. These samples suffer secondary minerals, such as smectite, montmorillonite, chlorite, and serpentine minerals, which are generally with elevated fluid-mobile element contents. In addition, relatively more evolved rocks of the Marianas show consistent differentiation trend with thin arcs, further suggesting that the scattered outlier is due to the extreme alteration effects.

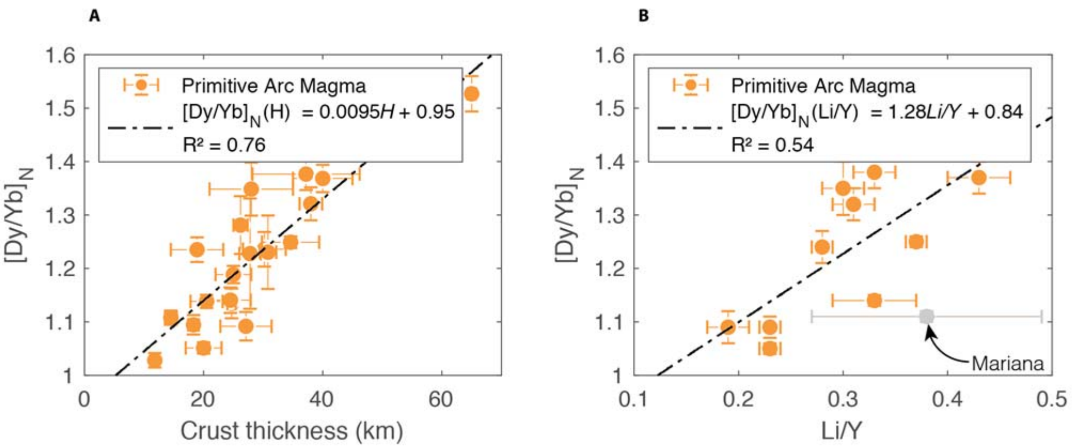

Supplementary Figure 2. Chondrite[3] normalized Dy/Yb in primitive arc magmas versus crustal thickness and Li/Y in primitive arc basalts of individual arc segments. Primitive arc magmas are related only to the samples with SiO_2 between 48-52 wt. % and MgO between 6-15 wt.%. In (B), the dull gray symbol represents the Marianas, which is with limited sample size and extensive alteration. Error bars for element ratio are 2se; error bars for crustal thickness are one standard deviation (1σ). Dashed lines in (A) and (B) represent linear regressions of the data with the Mariana arc excluded. The increasing Dy/Yb is the existing signature of garnet because of its preference for heavy rare earth elements (HREEs)[4,5]. Thereby, mantle melting in the presence of residual garnet will result in elevated Dy/Yb in magmatic melts. With crustal thickening and hence increasing melting pressure, we should expect progressively rising Dy/Yb as a consequence of the residual high pressure-favored garnet (Panel A). The close correlation of Li/Y with Dy/Yb in Panel B suggests that the subtle positive correlation between crustal thickness and Li/Y in primitive arc magma is imparted by residual mantle garnet.

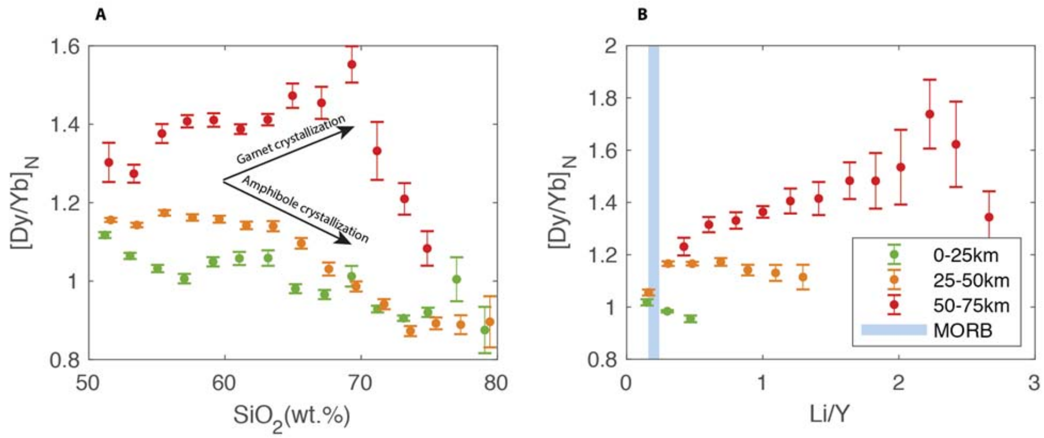

Supplementary Figure 3. Chondrite[3] normalized Dy/Yb versus SiO_2 and Li/Y in arc lavas for various crustal thickness. The errors are 2se. Pale blue line in (B) denotes the Li/Y ratio of MORB[6]. Dy/Yb ratio is a characterized indice for fractionation between middle rare earth elements (MREEs) and heavy rare earth elements (HREEs), which is dictated by crystallization of garnet and amphibole, with the former preferentially incorporating HREEs and thus increasing Dy/Yb, and the latter favorably sequestering MREEs and hence decreasing Dy/Yb[4,5]. Consistent paces between Dy/Yb and Li/Y during differentiation indicate that Li/Y fractionation is the consequence of saturation in garnet and amphibole.

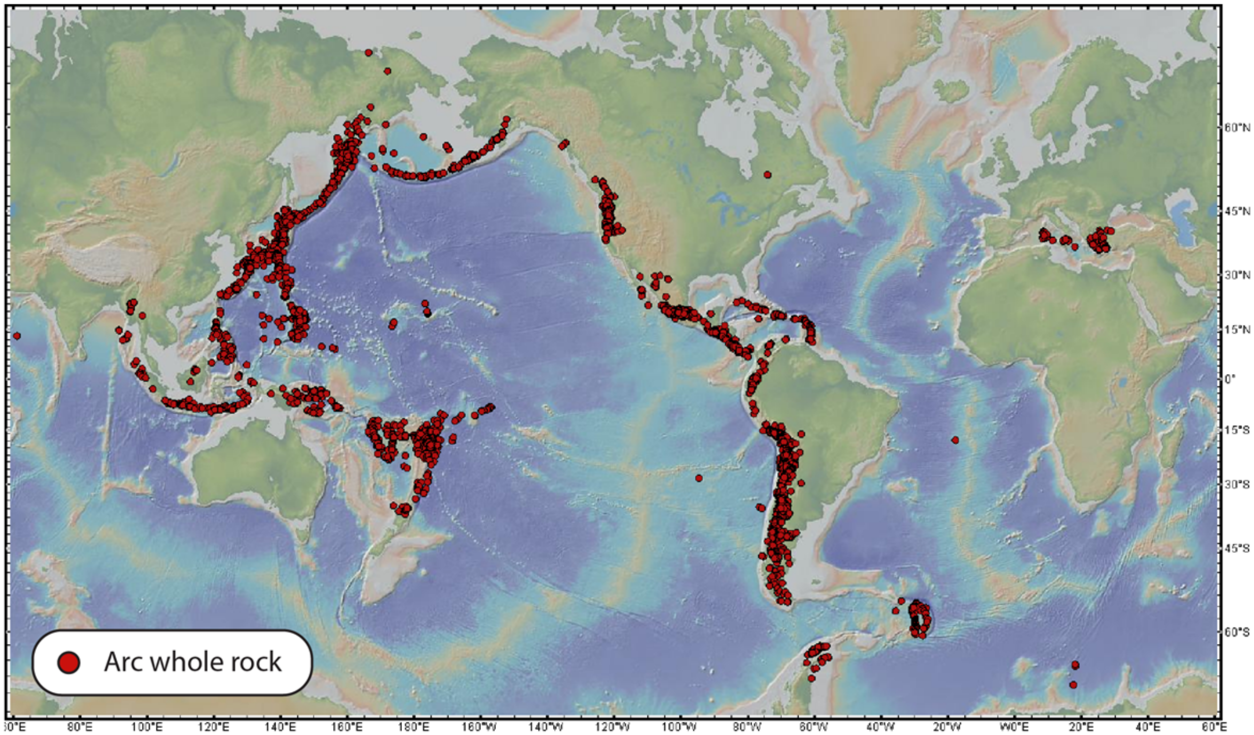

Supplementary Figure 4. Sample distribution of the arc whole rocks compiled in this study. Arc data are extracted from GEOROC [7]. The map was processed by GeoMapApp (http://www.geomapapp.org)[8].

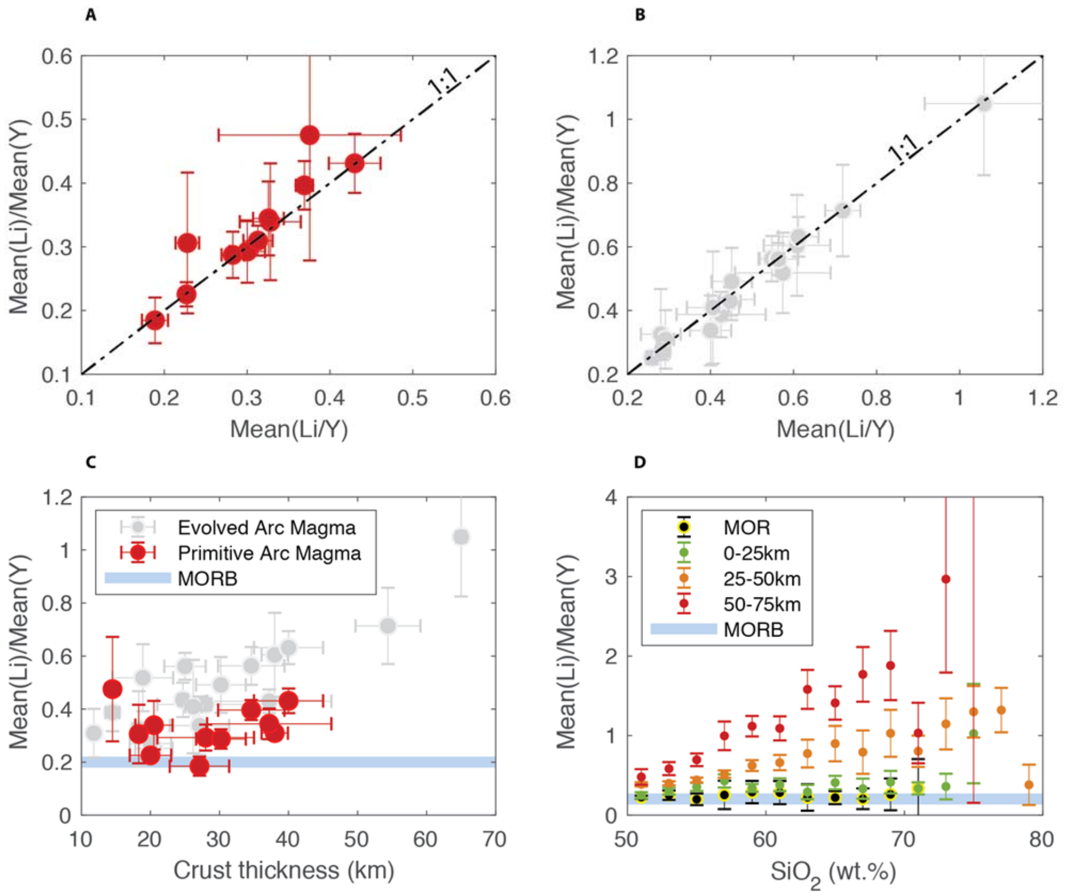

Supplementary Figure 5. Comparison of ratioing schemes. (A and B) Mean(Li)/Mean(Y) versus Mean(Li/Y) in primitive and evolved arc magmas. Mean(Li)/Mean(Y) represents Li/Y ratios calculated by ratioing the average values of Li and Y; Mean(Li/Y) corresponds Li/Y ratios calculated by directly averaging sample Li/Y ratios. Evolved arc magmas represent lava samples with 53-57 wt. % SiO_2. **Mean(Li)/Mean(Y) in primitive arcs versus crustal thickness (C) and Mean(Li)/Mean(Y) in arc magmas versus SiO_2 (D).** Error bars for Li/Y ratio are 2se; error bars for crustal thickness are 1σ. Pale blue lines are from Ryan and Langmuir [6]. Mid-ocean ridge data are extracted from Keller et al. [9].

Supplementary Reference:

1. Dietrich, V., Emmermann, R., Oberhänsli, R. & Puchelt, H. Geochemistry of basaltic and gabbroic rocks from the West Mariana Basin and the Mariana Trench. *Earth and Planetary Science Letters* **39**, 127-144 (1978).
2. Sharaskin, A. Y. PETROGRAPHY AND GEOCHEMISTRY OF BASEMENT ROCKS FROM 5 LEG-60 SITES. *Initial Reports of the Deep Sea Drilling Project* **60**, 647-656 (1982).
3. Sun, S.-S. & McDonough, W. F. Chemical and isotopic systematics of oceanic basalts: implications for mantle composition and processes. *Geological Society, London, Special Publications* **42**, 313-345 (1989).
4. Davidson, J., Turner, S. & Plank, T. Dy/Dy*: variations arising from mantle sources and petrogenetic processes. *J Petrol* **54**, 525-537 (2013).
5. Rudnick, R. & Taylor, S. Geochemical constraints on the origin of Archaean tonalitic-trondhjemitic rocks and implications for lower crustal composition. *Geological Society, London, Special Publications* **24**, 179-191 (1986).
6. Ryan, J. G. & Langmuir, C. H. The Systematics of Lithium Abundances in Young Volcanic-Rocks. *Geochim Cosmochim Ac* **51**, 1727-1741, doi:Doi 10.1016/0016-7037(87)90351-6 (1987).
7. GEOROC. *GEOROC*, <http://georoc.mpch-mainz.gwdg.de/georoc/> (
8. GeoMapApp. *GeoMapApp*, <http://www.geomapapp.org> (
9. Keller, C. B., Schoene, B., Barboni, M., Samperton, K. M. & Husson, J. M. Volcanic-plutonic parity and the differentiation of the continental crust. *Nature* **523**, 301-307, doi:10.1038/nature14584 (2015).
