# Supplementary material for: Lithium systematics in global arc magmas and the importance of crustal thickening for lithium enrichment
Source: Nat Commun. 2020 Oct 20;11:5313. doi: 10.1038/s41467-020-19106-z (PMC7575555; doi:10.1038/s41467-020-19106-z)
Supplement: Supplementary file 3 — Description of Additional Supplementary Files [file 41467_2020_19106_MOESM3_ESM.pdf]

Description of Additional Supplementary Files

File Name: Supplementary Data 1
Description: Compilation of lithium resources and reserves in 2019.

File Name: Supplementary Data 2
Description: Li and Li/Y statistics for individual arc segments; Generalized crustal thicknesses for specific arc segments; Slab thermal parameters extracted from Syracuse et al.

File Name: Supplementary Data 3
Description: Compiled dataset for global arc whole rocks.

File Name: Supplementary Data 4
Description: Comparison between different methods of calculating elemental ratios.

File Name: Supplementary Data 5
Description: Starting composition input for Rhyolite-MELTS simulation.

File Name: Supplementary Data 6
Description: Conditions for Rhyolite-MELTS simulation.
